# Co-modified 3D printed β-tricalcium phosphate with magnesium and selenium promotes bone defect regeneration in ovariectomized rat

**DOI:** 10.1007/s10856-022-06708-w

**Published:** 2023-01-09

**Authors:** Zhou-Shan Tao, Tian-Lin Li, Shan Wei

**Affiliations:** 1 Department of Trauma Orthopedics, The First Affiliated Hospital of Wannan Medical College, Yijishan Hospital, No. 2, Zhe shan Xi Road, Wuhu, 241001 Anhui People’s Republic of China; 2 School of Mechanical Engineering, Anhui Polytechnic University, Wuhu, 241000 P.R. China; 3 Additive Manufacturing Institute of Anhui Polytechnic University, Anhui Polytechnic University, Wuhu, 241000 P.R. China

## Abstract

Magnesium (Mg) and Selenium (Se) are essential elements for bone health and have been studied extensively for its powerful osteogenesis and promoting bone regeneration. The purpose was to observe whether Co-modified 3D-printed β-tricalcium phosphate with Mg and Se could promote bone defect regeneration in an ovariectomized(OVX) rat model. The MC3T3-E1 cells were co-cultured with the leachate of β-TCP, Mg-TCP, and Mg/Se-TCP and induced to osteogenesis, and the cell viability, ROS, and osteogenic activity were observed by Cell Count Kit-8(CCK-8), fluorescent probe 2′, 7′-dichlorofluorescin diacetate, Alkaline phosphatase (ALP) staining, Alizarin Red(RES) staining, western blotting(WB), and immunofluorescence. Then the β-TCP, Mg-TCP, and Mg/Se-TCP were implanted into the femoral epiphysis bone defect model of OVX rats for 12 weeks. Micro-CT and histology analysis were used to observe the therapeutic effect. In vitro results show that the cell mineralization and osteogenic activity of the Mg/Se-TCP group is significantly higher than the β-TCP group and Mg-TCP group. Protein expressions such as FOxO1, SIRT1, SOD2, Runx-2, Cola1a, and OC of the Mg/Se-TCP group are significantly higher than the Con group and the β-TCP group. The results of intracellular ROS and SIRT1 and SOD2 immunofluorescence showed that Mg/Se-TCP can restore the oxidative stress balance of osteoblasts. Micro-CT and histology analysis showed that treatment with Mg/Se-TCP showed the largest amount of bone tissue in the defect area (*p* < 0.05), and exhibited lower values of residual biological material (*p* < 0.05), compared to that of the β-TCP group and Mg-TCP group. Our research results confirm that Mg/Se-TCP can improve the activity and function of osteoblasts and enhance bone regeneration mediated by reducing intracellular ROS in OVX rat models.

**Graphical Abstract:**

**Figure:**
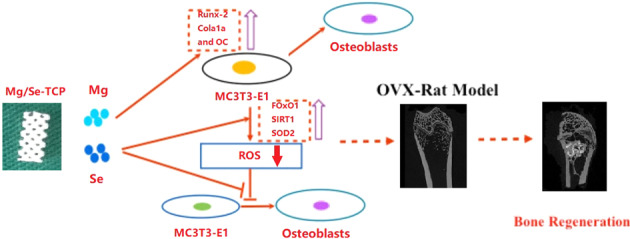
The release of Mg and Se during the degradation of Mg/Se-TCP can improve the local bone repair ability. At the same time, it can also inhibit cell ROS, and ultimately greatly promote local bone repair.

## Introduction

Autologous bone graft is a successful orthopedic intervention for nonunion of fracture and bone defect, which relieves pain, restores function and good results, and improves the overall quality of life [1]. With an increase in aging population and metabolic bone diseases, the number of nonunion of fractures and bone defects in elderly patients has also increased [2]. In recent years, the need for autologous bone graft surgery due to bone nonunion and the bone defect has brought both challenges to patients and clinical orthopedic surgeons for limited donor bone and increased surgical site [3]. Excessive bone resorption with limited bone formation could lead to bone loss and a fast decrease in bone mass, which could cause a reduction in the impaired bone repair capacity and limited bone regeneration [4], especially in renal osteodystrophy, postmenopausal osteoporosis, and diabetic osteoporosis. The development of osteoporosis in the elderly population and postmenopausal women results in decreased ability to repair, including bone loss, fractures delayed union or nonunion [5, 6]. As the osteoporotic population continues to grow and becomes an increasingly serious global public health problem, how to reduce fracture and bone defect treatment failures and improve bone repair in osteoporotic patients has become a problem that demands prompt solutions from clinicians and academics [7, 8].

With the advancement of cell-based bone tissue engineering, the appearance of beta-tricalcium phosphate (β-TCP) scaffold generates a promise to break the “Gold Standard” [9, 10]. Owing to the needed characteristics of similar components, biocompatibility and biodegradability, β-TCP bioceramics are already applied clinically for a variety of orthopedic applications [10, 11]. In recent years, with the development of manufacturing technologies, 3D-printing has become a major driver for material structure innovation [12–14]. Although 3D-printed β-TCP has better performance than traditional β-TCP, currently available β-TCP has limited efficacy due to a lack of sufficient osteoinductive activity, especially in the osteoporotic state [12–14]. It is well known that biomaterials with beneficial ions such as zinc, manganese, magnesium, strontium, and selenium alter the tissue microenvironment and are conducive to bone healing and regeneration [15, 16]. Selenium(Se), an essential and important element in a variety of physiological processes, has antioxidant, anti-inflammatory and enhancing osteogenic differentiation of bone marrow-derived mesenchymal stem cells (MSCs) [17, 18]. Recent studies have found that local administration with Se could induce bone formation and promote angiogenesis, and could improve bone regeneration in an osteoporosis rat model [19, 20]. As a metal ion with bone-seeking properties, Magnesium (Mg) is described as an important trace element in bone [21, 22]. Some scholars found that Mg promotes osteoblast differentiation and osteogenesis [12, 16, 21, 22]. Our previous study has shown that local use of Mg promotes titanium rod osseointegration in osteoporotic rats [16].

Since both Mg and Se can contribute to enhanced osteogenesis and Se also inhibits osteoclast formation and reduces osteoclast activity [23], treatment of bone defect with both of these factors combined may enhance bone formation more effectively than did either treatment alone. Moreover, improvement osteogenesis and suppress osteoclastic activities simultaneously, can achieve a better curative effect for bone regeneration [24], suggesting the possibility of a synergistic effect between Mg and Se. However, it has not been reported whether local use of both Mg and Se has a potentially beneficial effect on (β-TCP -mediated bone defect regeneration in OVX rats. Therefore, in our current research, we propose the following hypotheses that Mg and Se relies on its excellent osteoinductive ability to promote the proliferation and differentiation of osteoblasts, thereby enhancing the osteogenic function in the state of osteoporosis, finally changing the performance of poor regeneration in osteoporotic rats. In this study, we explored the effect of Mg-Se modified β-tricalcium phosphate-mediated bone defect regeneration with an established osteoporotic rat model, and initially explored possible mechanisms.

## Materials and methods

### Animals and reagents

Healthful female Sprague Dawley non-breeder rats, 3 months of age and 228.3 ± 12.5 g in body weight, were included in the present study. The animals were housed under a standard experimental environment with a 12/12-h light/dark cycle and free access to a standard diet and water. Calcium hydrogen phosphate dihydrate (CaHPO_4_·2H_2_O) and calcium carbonate (CaCO_3_), MgO, SeO_2_, gelatin, and absolute ethanol were purchased from Shanghai Aladdin Bio-Chem Technology Co., Shanghai, China. All the commercial chemicals are analytically pure. Cell, cell-associated complete culture media, reagents, and antibodies for cell experiments will be described later.

### 3D-printing preparation of β-TCP scaffolds, Mg-modified β-TCP scaffolds(Mg-TCP), and Mg-Se modified β-TCP scaffolds(Mg/Se-TCP)

First, β-TCP, Mg-TCP, and Mg/Se-TCP powder need to be prepared as previously described [25]. Briefly, the β-TCP powder was synthesized with CaHPO_4_·2H_2_O and CaCO_3_ under a high-temperature solid phase reaction method. It should be pointed out that the mixing ratio of CaHPO_4_·2H_2_O and CaCO_3_ powders is Ca/P = 1.5. The M-TCP, S-TCP, and MS-TCP powders were a mixture of β-TCP and MgO or/and SeO_2_ powders at various ratios. Two groups of Mg-TCP and Mg/Se-TCP powders were prepared with MgO of 3 wt% and MgO of 3 wt% plus SeO_2_ of 2 wt%. Afterwards, β-TCP, Mg-TCP, and Mg/Se-TCP scaffolds(2.5 mm in diameter and 6 mm in height) were prepared through a 3D-printing device (Livprint Norm, Medprin, Guangzhou, China) and sintered at 1100 °C for 3 h as previously described [25]. Briefly, 4 g of β-TCP, Mg-TCP and Mg/Se-TCP powder and 2 mL 20 wt% gelatin solution (20 wt% dry gelatin basis) were added to a 10 mL beaker to prepare the β-TCP, Mg-TCP and Mg/Se-TCP bio ink. The powder-gel mixture was thoroughly stirred with a glass rod until it looked like toothpaste. Briefly, the powder-gel mixture was loaded into a special 3 ml syringe, and a printing nozzle having an inner diameter of 420 lm (22 gauge) was used in consideration of suitable filament diameter and pore diameter. The temperature of the platform was set to − 5 °C to ensure rapid solidification. After all the printed scaffolds were freeze-dried for 48 h, they were sintered at 1250° for 2 h in a muffle furnace to remove the organic binder.

### Detection and evaluation of scaffolds characterization

Field-emission scanning electron microscopy (SEM, S-4800, Hitachi, Japan) and energy-dispersive X-ray spectrometry (EDS, Oxford, IE250, UK) were used to observe the surface morphology and the elemental mapping of β-TCP scaffolds, Mg-TCP scaffolds, and Mg/Se-TCP scaffolds. X-ray diffractometry (XRD, Empyrean PANalytical Netherlands) and surface roughness tester (Mitutoyo SJ-400, Mitutoyo, Sakado, Japan) were used to observe the phase composition and surface roughness of this scaffolds. Mercury intrusion porosimetry (MIP, AutoPore IV 9500, Micromeritics) and surface area analyzer (Gemini VII 2390t, Micromeritics) were used to observe the porosity and specific surface area of this scaffolds.

### Animal experiments

**Fig. 1:**
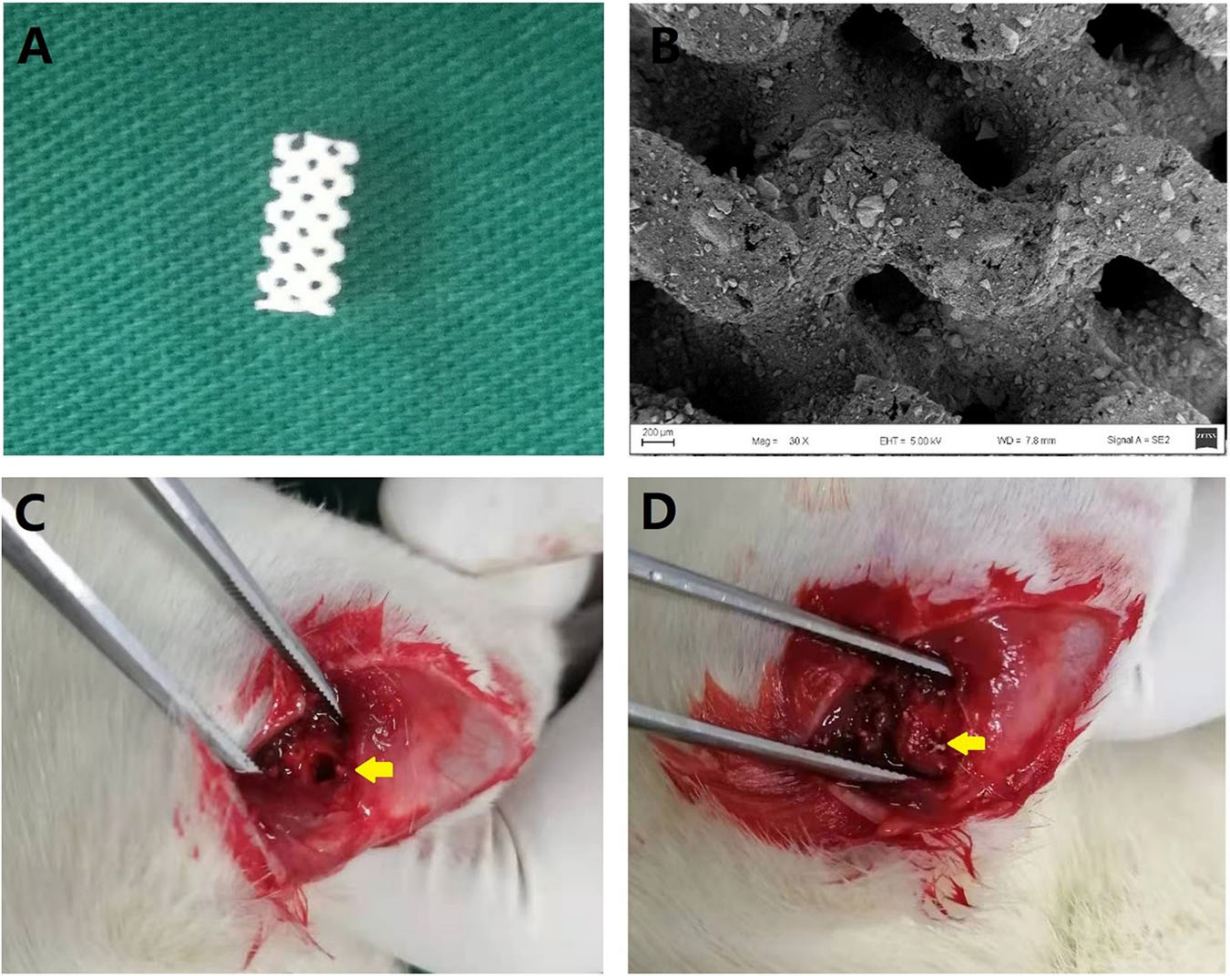
The 3D-printed bioscaffolds was implanted into the bone defect at the femoral metaphysis in ovariectomized rats. **A** The general view of the 3D-printed bioscaffolds(2.5 mm diameter and 6 mm height); **B** Electron microscope observation of the microstructure of the 3D-printed bioscaffolds (×30); **C** The bone defect model at femoral metaphysis; **D** Bioscaffolds implantation in the defect area; the yellow arrow indicates the location of the defect and the site where the biomaterial is implanted

The osteoporosis model was established by bilateral ovariectomy for 12 weeks according to a previously-described protocol [26, 27]. Next, these animals were randomly divided into four groups of ten rats each: OVX rats group(OVX), OVX defected rats receiving β-TCP treatment group(β-TCP), OVX defected rats receiving Mg-TCP treatment group(Mg-TCP), and OVX defected rats receiving Mg/Se-TCP treatment group(Mg/Se-TCP). Then, the scaffolds were inserted with a drilling bone defect with a 3 mm external diameter bilaterally in all animals from a β-TCP group, Mg-TCP group and Mg/Se-TCP group as previously described [28, 29]. The rats were classified into β-TCP, Mg-TCP, and Mg/Se-TCP groups and were implanted with β-TCP, Mg-TCP, and Mg/Se-TCP scaffolds as shown in Fig. 1, respectively. The deadline for an experiment after implant surgery continued for a total course of 12 weeks, the femur of living rats were harvested and evaluated by histological examination, biomechanical testing, biochemical testing, and Micro-CT scanning and analysis. All experiments were approved and carried out in accordance with international standards on animal welfare and the Institutional Animal Care Committee of Wannan Medical College (Approval No. LLSC-2020-082).

### Micro-CT evaluation

The right distal femur containing bioscaffolds were repeatedly (12 weeks after treatment) scanned by SkyScan 1176 in vivo micro-computed tomography (Micro-CT, Bruker, Kontich, Belgium) with 10-μm voxel size as previously described [30, 31]. The scanning parameters were set with 55 kV and 114 mA, 1024 reconstruction matrix, and 200 ms integration time, a thickness of 0.048 mm per slice in medium-resolution mode. Trabecular bone microarchitecture was scanned and performed on 100 slices (0.9 mm total) with a distance of 2 mm proximal to the growth plate in the femoral metaphysis. By drawing a volume of interest (VOI) with the central 2.5-mm-diameter region of the center of the bone defect, the newly formed bone tissue could be defined separately for the edge of the bone defect. After 3D reconstruction, BMD, BV/TV, Tb.N, Tb.Th, Tb.Sp, Conn.D in VOI regions were used to assess bone regeneration and detect bone formation at the site around the bone defect.

### HE staining and immunohistochemical analysis

These femurs were incubated in 10% EDTA (pH 7.4) at 4 °C for 4 weeks before paraffin-embedding. The longitudinally oriented bone defect sections with 4-µm-thick were collected for HE staining and immunohistochemical staining as previously described [19, 32]. In brief, histological examination of decalcified sections were performed by staining with HE for light microscopy. Immunohistochemical staining of tartrate-resistant acid phosphatase-5b(TRACP-5b) and osteocalcin (OC) were used to assess local bone remodeling. The sections of the defected area were irrigated and incubated overnight with the commercially-available specific antibodies TRACP-5b (1:100, Abcam, Cambridge, UK) and OC (1:100, Abcam, Cambridge, UK) at 4 °C. Then, the slides were incubated with the corresponding goat anti-rabbit secondary antibody for 30 min and counter-stained with diaminobenzidine and hematoxylin. Finally, the images of bone tissue after different interventions were observed and obtained by using a light microscope.

### MC3TE-E1 cell experiments

In the cell experiment, the intervention liquid, including β-TCP, Mg-TCP, and Mg/Se-TCP were the leaching solution which were produced by β-TCP, Mg-TCP, and Mg/Se-TCP scaffolds in PBS for 24 h. MC3TE-E1, an immortalized and murine osteoblast cell line purchased from Qingqi (Shanghai) Biotechnology Development Co., Ltd.), was used in this study in vitro. Initially, MC3TE-E1 Cells (1 × 10^4^ cells/mL) were seeded in different cell culture environment as follows: phosphate-buffered saline (PBS, Con group), the extract of β-TCP scaffold (20 μmol/l, β-TCP group), Mg-TCP scaffold (20 μmol/l, Mg-TCP group), and Mg/Se-TCP scaffold (20 μmol/l, Mg/Se-TCP group). The cell culture media used in this study was Dulbecco’s modified Eagle’s medium (DMEM, Rockville, MA, USA). Cell Counting Kit-8 (CCK-8), alizarin Red S (RES) staining, and alkaline phosphatase (ALP) staining were used to determine the effects of SIL and high glucose on MC3TE-E1 cell’s biological characteristics, including changes in the activity, function, intracellular ROS levels and the expression of intracellular target proteins.

Briefly, MC3TE-E1 was seeded in a 96-well plate with a density of 1 × 10^4^ cells per well and treated with PBS and the different extracts of scaffolds as mentioned above for 24, 48, and 72 h. Subsequently, the cells from different groups after intervention were added with 10-μl CCK-8(Med Chem Express LLC; Monmouth Junction, NJ, The USA) solution for 2 more hours and evaluated through a Multiskan Go Microplate Spectrophotometer (Thermo Fisher Scientific). Meanwhile, the fluorescent probe 2′, 7′-dichlorofluorescin diacetate(DCFDA) was performed to measure the production of total intracellular ROS from MC3TE-E1 under H_2_O_2_(400 μM) and different interventions according to the previous report [19]. Then, immunofluorescence staining was performed to determine the protein expression of SIRT1 and SOD2 of MC3TE‐E1 after different interventions as described above.

For RES staining and ALP staining, MC3TE-E1 was seeded in a 96-well plate with a density of 1 × 10^4^cells per well and treated with osteogenic medium (complete α-MEM containing 1 nM dexamethasone, 50 μM ascorbic acid, and 20 mM β-glycerophosphate) [33] before reaching over 80% confluence. Next, the MC3TE-E1 cells receive interventions with different treatment options, as mentioned above. After treatment for 14 days, osteogenesis was assessed using ALP (Beyotime Institute of Biotechnology; Jiangsu, China) staining. After treatment for 21 days, osteogenic differentiation and cell mineralization was detected using RES solution (Solarbio Science & Technology). The results of ALP staining and RES staining were determined by microscopy and quantified with Image Pro Plus 6.0 software.

### Western blot

The extracts of MC3TE-E1 for western blotting were prepared after the indicated treatment for 3 days as previously described [34]. About 20 μg protein from cells culture lysates were harvested using PRO-PREPTM protein extraction solution (Boca Scientific Inc., Boca Raton, FL) and carried out subsequent electrophoresis, according to the manufacturer’s instruction. The primary antibodies against the following proteins: FoxO1 (Abcam, 1:1000), SIRT1 (Abcam, 1:1000), superoxide dismutase 2 (SOD2, Abcam, 1:1000), Runx-2 (Abcam, 1:1000), OC (Abcam, 1:1000), and Cola1a (Abcam, 1:1000). Expression levels of the target protein were normalized against glyceraldehyde 3-phosphate dehydrogenase (GAPDH) (Boster, Wuhan, China, 1:2000) levels in each sample. The following day, HRP-conjugated goat anti-rabbit, which was used as secondary antibodies, was purchased from Santa Cruz Biotechnology (Santa Cruz, CA) and the results were detection and analysis by using an iBrightCL1000 (Invitrogen, Carlsbad, CA).

### Statistical analysis

The experimental results were expressed as mean ± standard deviation. The experimental data were analyzed using SPSS (19.0) statistical software, and One-way analysis of variance (ANOVA) was used for multiple between-group comparisons followed by Tukey’s post hoc test. The independent t-test was used for comparisons of the Sham group and the OVX group. A value of *P* < 0.05 was considered to reflect significance.

## Results

### Characterization of the bioscaffolds

**Fig. 2:**
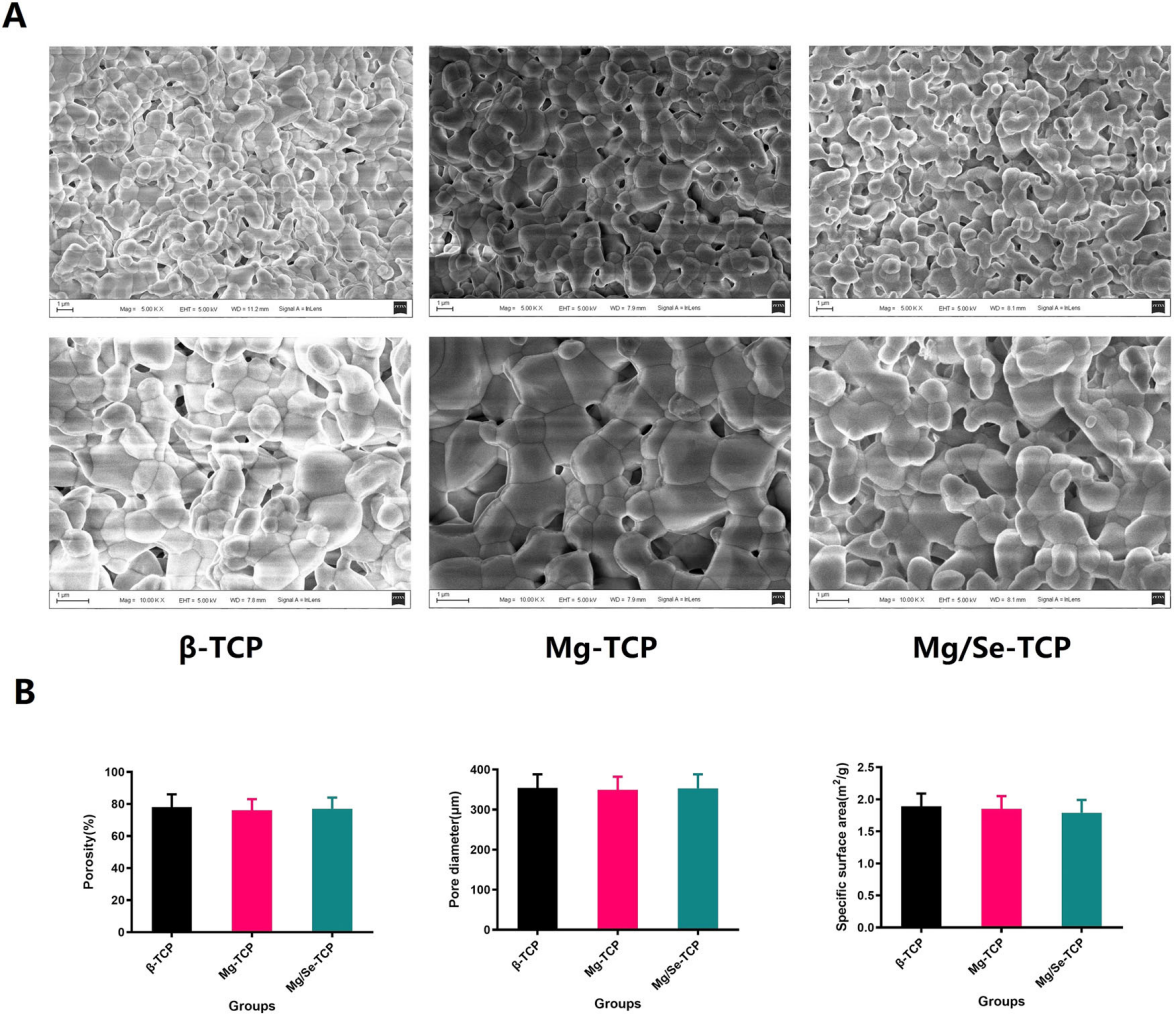
Physiochemical properties of the β-TCP, Mg-TCP, and Mg/Se-TCP constructed by 3D-printing. **A** Scanning electron images of the β-TCP, Mg-TCP, and Mg/Se-TCP. **B** The porosity, mean pore diameter, and specific surface area of the β-TCP, Mg-TCP, and Mg/Se-TCP

Figure 2A displayed the surface morphology of β-TCP, Mg-TCP, and Mg/Se-TCP and the porosity, mean pore diameter, and specific surface area of these bioscaffolds. Highly porous structure and interconnected pore size were observed from β-TCP, Mg-TCP, and Mg/Se-TCP with 300–400 μm and ~100 μm, respectively. Quantitative results, including the porosity, mean pore diameter and specific surface area of the β-TCP, Mg-TCP, and Mg/Se-TCP were observed with no differences (*p* = 0.67; Fig. 2B).

**Fig. 3:**
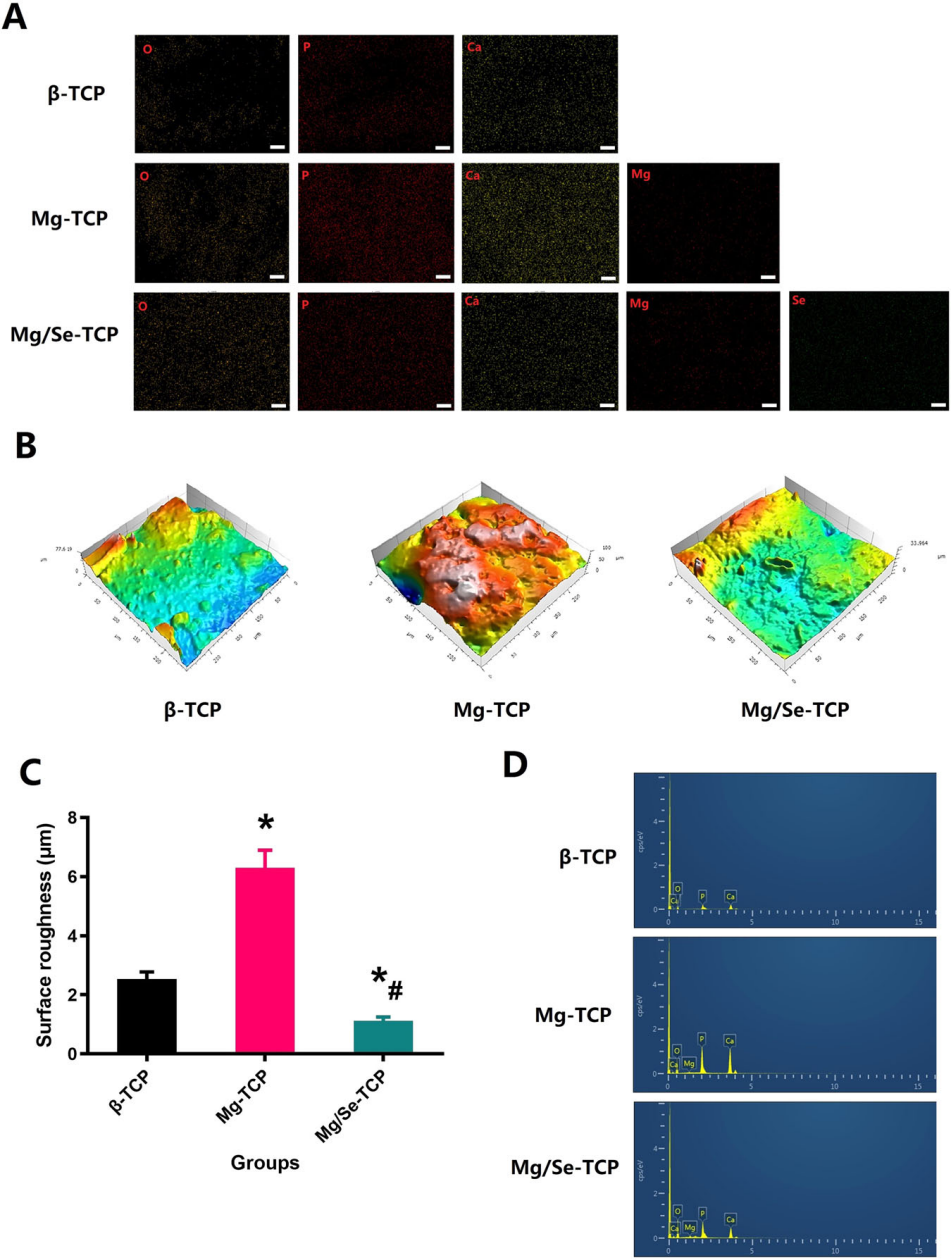
Phase composition analysis of the β-TCP, Mg-TCP, and Mg/Se-TCP. **A** EDS mapping for the major elements of the β-TCP, Mg-TCP, and Mg/Se-TCP. **B** The roughness of the β-TCP, Mg- TCP, and Mg/Se-TCP surfaces. **C** Quantitative results of the roughness of β-TCP, Mg- TCP, and Mg/Se-TCP bioscaffolds. **D** XPS spectra of the β-TCP, Mg- TCP, and Mg/Se-TCP bioscaffolds

Elemental mapping of β-TCP, Mg-TCP, and Mg/Se-TCP confirmed the presence and homogeneous distribution of Mg, Se, and other elements (Fig. 3A). Clearly, β-TCP was smoother than that of Mg-TCP, the mean surface roughness of the Mg-TCP sample was statistically greater than that of the β-TCP (Fig. 3B, C). However, a different tendency could be observed in the Mg/Se-TCP. After Se-coating the Mg-loaded TCP-based bioscaffold, the surface roughness was completely covered, and the morphology tended to be smoother. According to the XRD patterns (Fig. 3D), the spectra of β-TCP, Mg-TCP, and Mg/Se-TCP presented similar characteristic bands.

### Experimental animal

No animal death was found in the ovariectomy and sham surgery. A total of five rats died during or after building a bone defect model, including one rat in the OVX group, one rat in the β-TCP died 1 day after surgery and three rats in the Mg-TCP and Mg/Se-TCP group died 2 days after surgery.

### Osteoporosis animal model

**Fig. 4:**
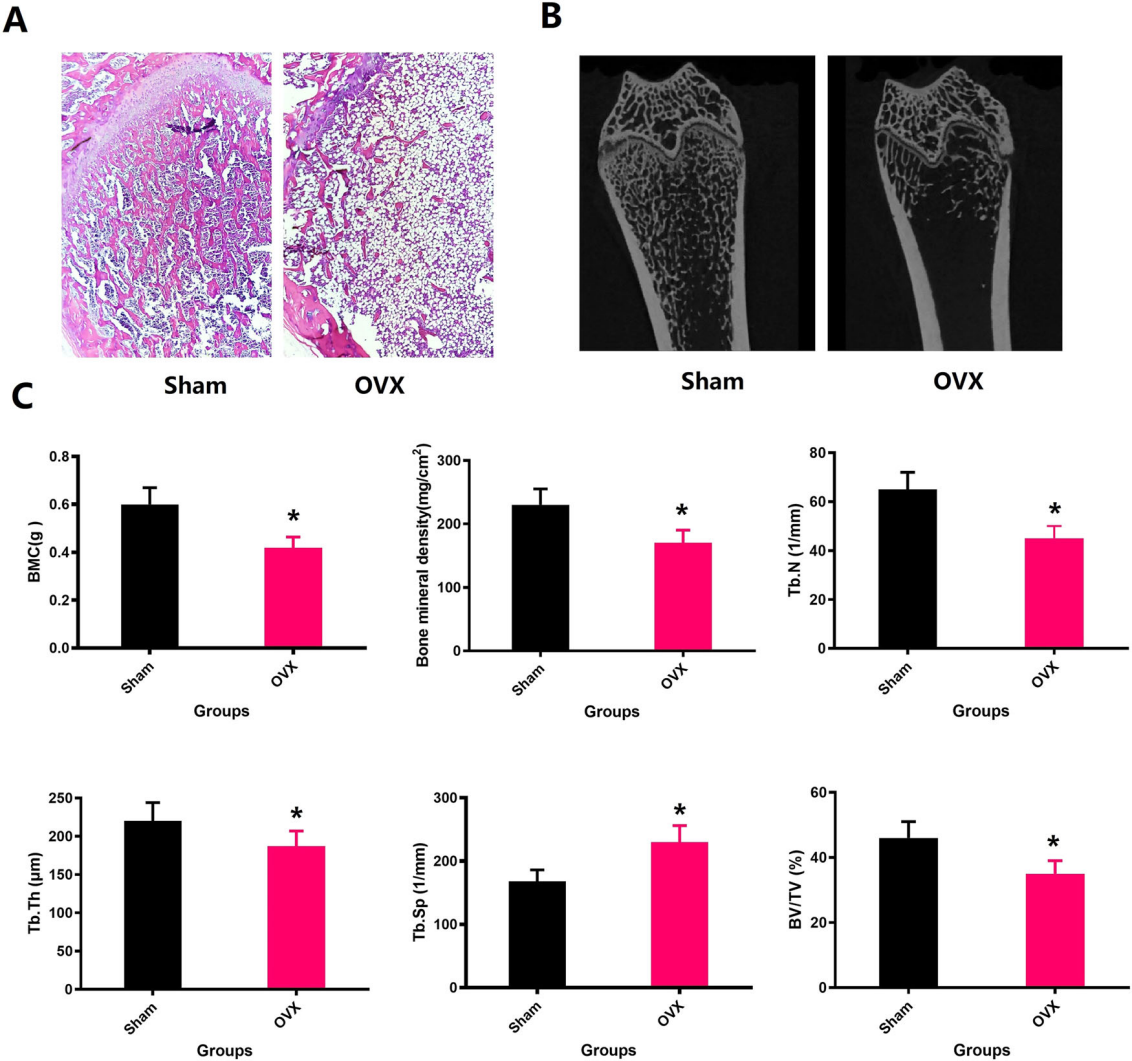
**A**, **B** The representative micro-CT sagittal 2D images(scale bar = 1 mm) and H&E staining images (magnification of 20) show that the trabecular bone volume of the femoral distal metaphysis was markedly reduced after bilateral ovariectomy for 12 weeks. **C** The trabecular bone quantitative parameters containing bone mineral content (BMC), bone mineral density (BMD), bone volume fraction (BV/TV), trabecular number (Tb.N), trabecular thickness (Tb.Th), and trabecular separation (Tb.Sp) from OVX group and sham group. *P < 0.05 versus the Sham group

Twelve weeks after the first operation, five representative rats in each group were sacrificed and the trabecular architecture of the rat femoral metaphysis were measured by Micro-CT and HE. Figure 4A clearly shows that the trabecular bone in the distal femoral metaphysis significantly decreased in the ovariectomized rats, indicating severe bone loss after surgery. The trabecular bone quantitative parameters such as BMD, BMC, BV/TV, Tb. Th, Tb. N, and Tb. Sp are presented in Fig. 4B and these indexes were detected with notable differences between the two surgical groups (*P* < 0.05). When observing HE sections under a microscope, it is easy to find the same results. These results indicate that a bilateral ovariectomy-induced osteoporotic rat model was successfully established.

### Micro-CT evaluation

**Fig. 5:**
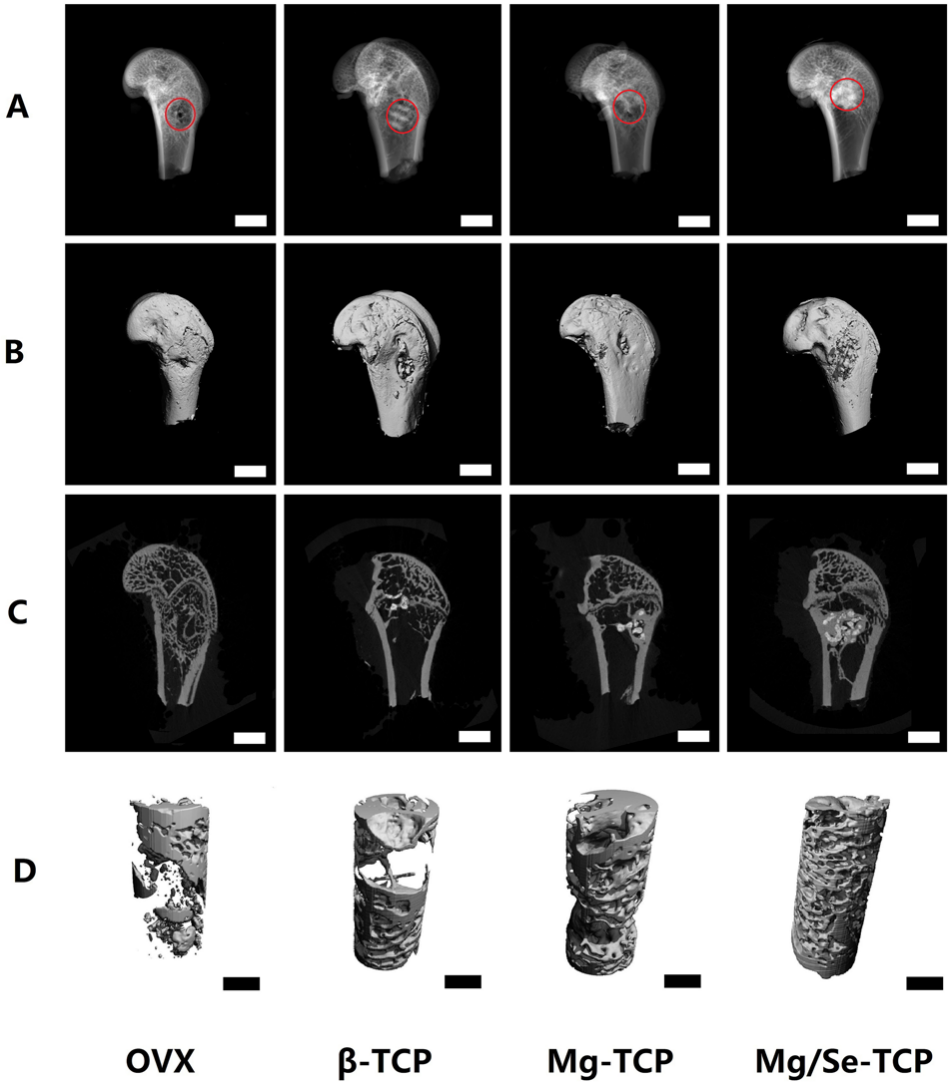
**A** X-ray detection; **B** Three-dimensional reconstruction of the overall view. **C** Three-dimensional CT scan; **D** 3D reconstruction of bone repair in the defect area

**Fig. 6:**
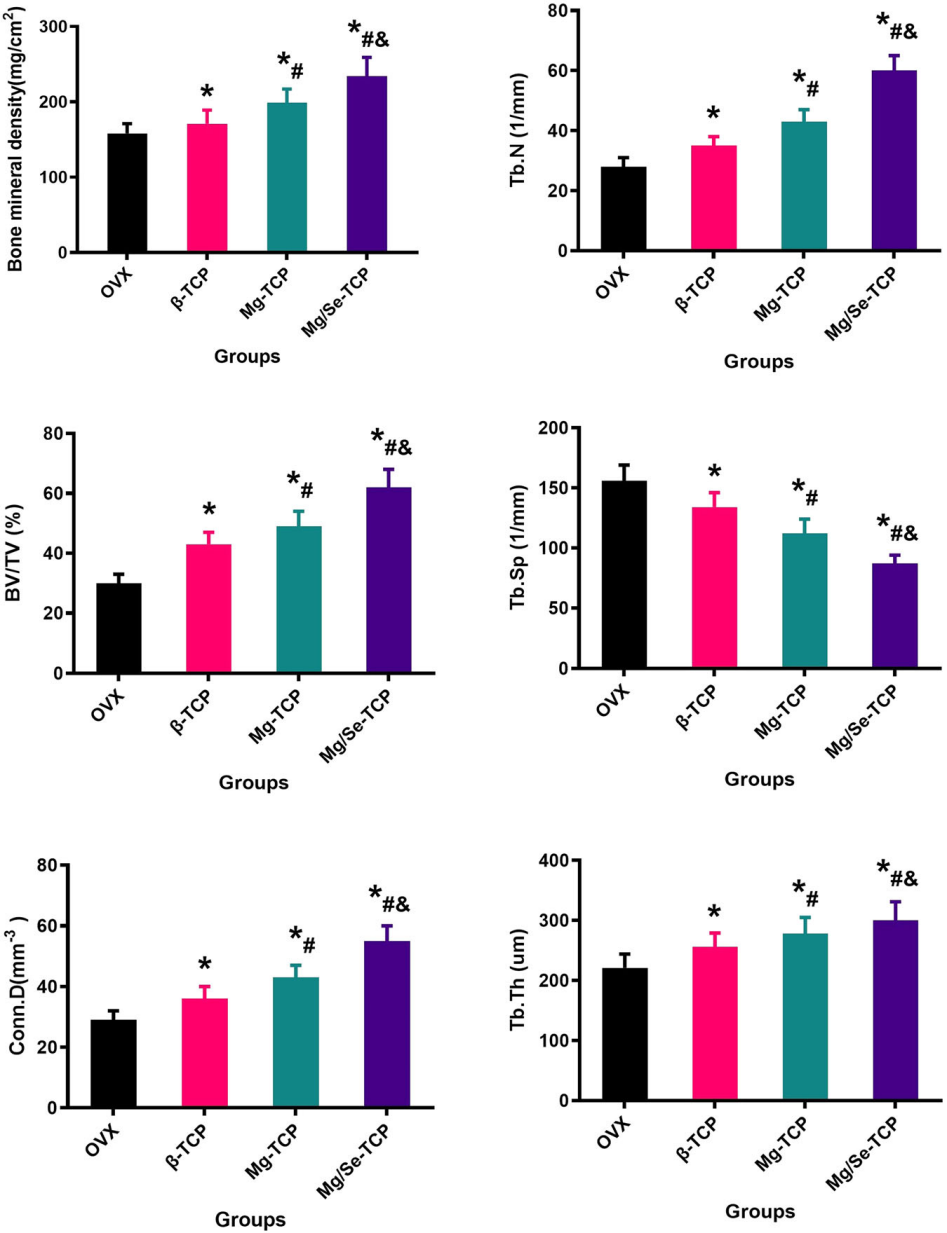
The quantitative parameters, including bone mineral density (BMD), bone volume fraction (BV/TV), trabecular number (Tb.N), trabecular thickness (Tb.Th), trabecular separation (Tb.Sp), the mean connective density (Conn.D).*Vs. OVX group, *p* < 0.05, ^#^Vs. β-TCP, *p* < 0.05, &Vs. Mg-TCP, *p* < 0.05

The results of bone repair for the bone defects in the femoral metaphysis from the four groups of rats with different interventions via Micro-ct evaluation are shown in Fig. 5. After local administration with Mg/Se-TCP for 12 weeks, the bone mass in the defected area was increased in the Mg/Se-TCP group compared with the β-TCP and Mg-TCP groups. Figure 6 illustrates the quantification of BMD, BV/TV, Tb. Th, Tb. N, Conn. D, and Tb. Sp among four groups. After Mg/Se-TCP treatment for 12 weeks, bone microscopic parameters of the Mg/Se-TCP group show higher BMD, BV/TV, Tb. N, Conn.D, Tb. Th, and the lower Tb. Sp (*P* < 0.05) compared with the β-TCP and Mg-TCP groups. Micro-CT 3D reconstruction and quantitative parametric results clearly demonstrate that Mg/Se-TCP treatment can significantly promote bone regeneration at bone defect sites in OVX rats.

### Histological and immunohistochemical analysis

**Fig. 7:**
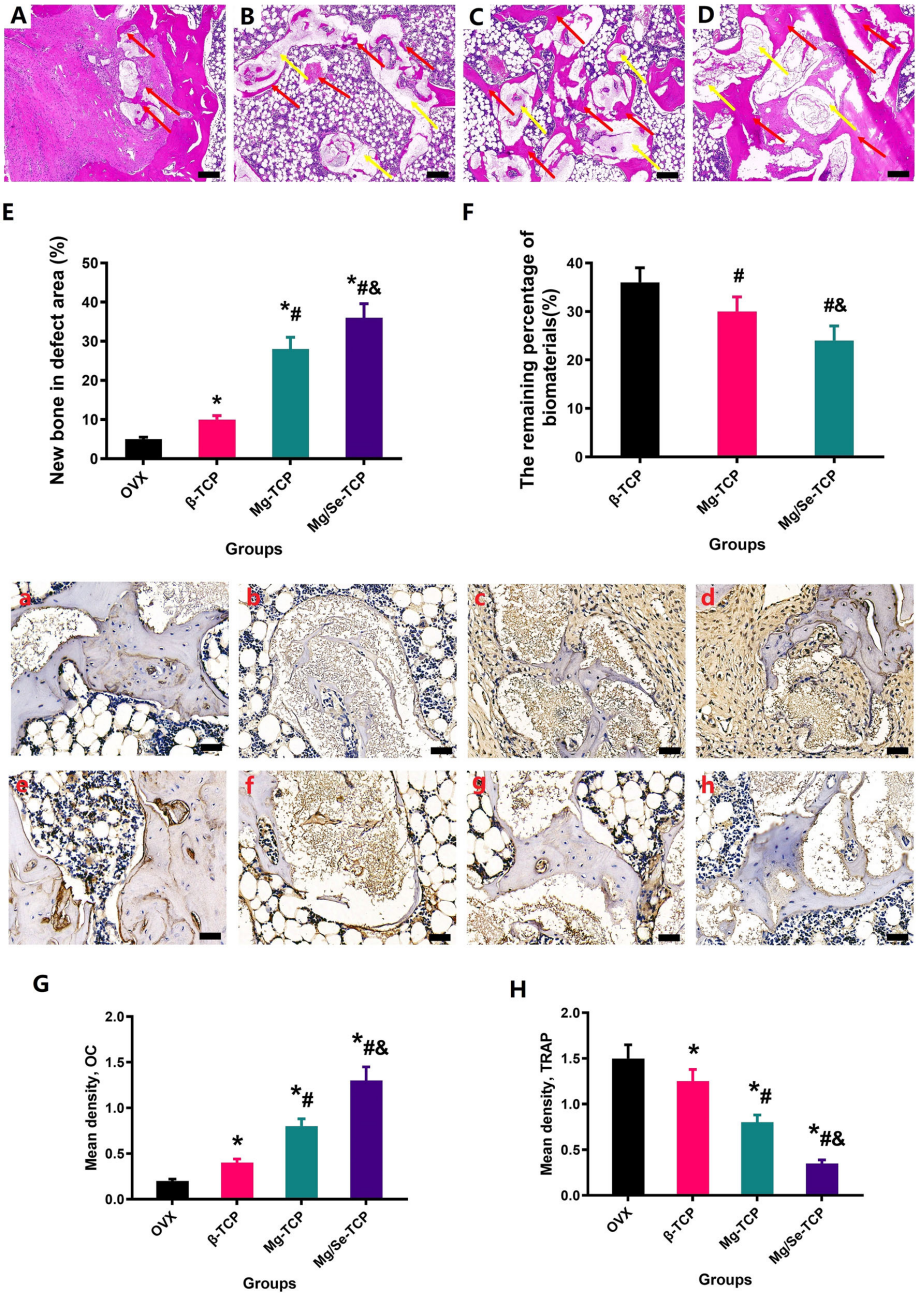
Bone regeneration was evaluated by histological (magnification, ×20) and quantitative analysis of bone repair and residual biological material (**E**, **F**) of the defected area in the OVX (**A**), β-TCP (**B**), Mg-TCP (**C**), and Mg/Se-TCP (**D**) groups. After treatment, the defective area was detected by OC staining (magnification, ×400) and TRACP-5b staining (magnification, ×400) in the OVX (**A**, **E**), β-TCP(**B**, **F**), Mg-TCP (**C**, **G**), and Mg/Se-TCP (**D**, **H**) groups. **G**, **H** The density of the OC-stained area and TRACP-5b-stained area was quantified. *n* = 5 per group. *Vs. OVX group, *p* < 0.05, ^#^Vs. β-TCP, *p* < 0.05, &Vs. Mg-TCP, *p* < 0.05

The results of bone repair for the bone defects in the femoral metaphysis from the four groups of rats with different interventions via histological evaluation are shown in Fig. 7A. In 12 weeks, large areas of the defective area still exist in the OVX group, with fewer new bone tissue around the defect. However, a large amount of quantity and maturity bone were observed filled in the defect area from the Mg-TCP group and the Mg/Se-TCP group. Meanwhile, a lot of biological material is still observed in the β-TCP group, while most of the Mg/Se-TCP group’s biological materials have degraded. In quantitative analysis, treatment with Mg/Se-TCP showed the largest amount of bone tissue in the defect area (*p* < 0.05), and Mg/Se-TCP treatment exhibited lower values of residual biological material (*p* < 0.05), compared to that of the OVX group, Mg-TCP group and the β-TCP group. The expression of OC was upregulated in the β-TCP group, Mg-TCP group, and Mg/Se-TCP groups. In addition, TRACP-5b staining showed an increase in the expression of TRACP-5b in OVX rats, which decreased after the administration of β-TCP, Mg-TCP, and Mg/Se-TCP. TRACP-5b-positive signals in the Mg/Se-TCP group were significantly lower than in the OVX group, β-TCP group, and Mg-TCP group.

### Osteogenic potential of Mg/Se-TCP in vitro

**Fig. 8:**
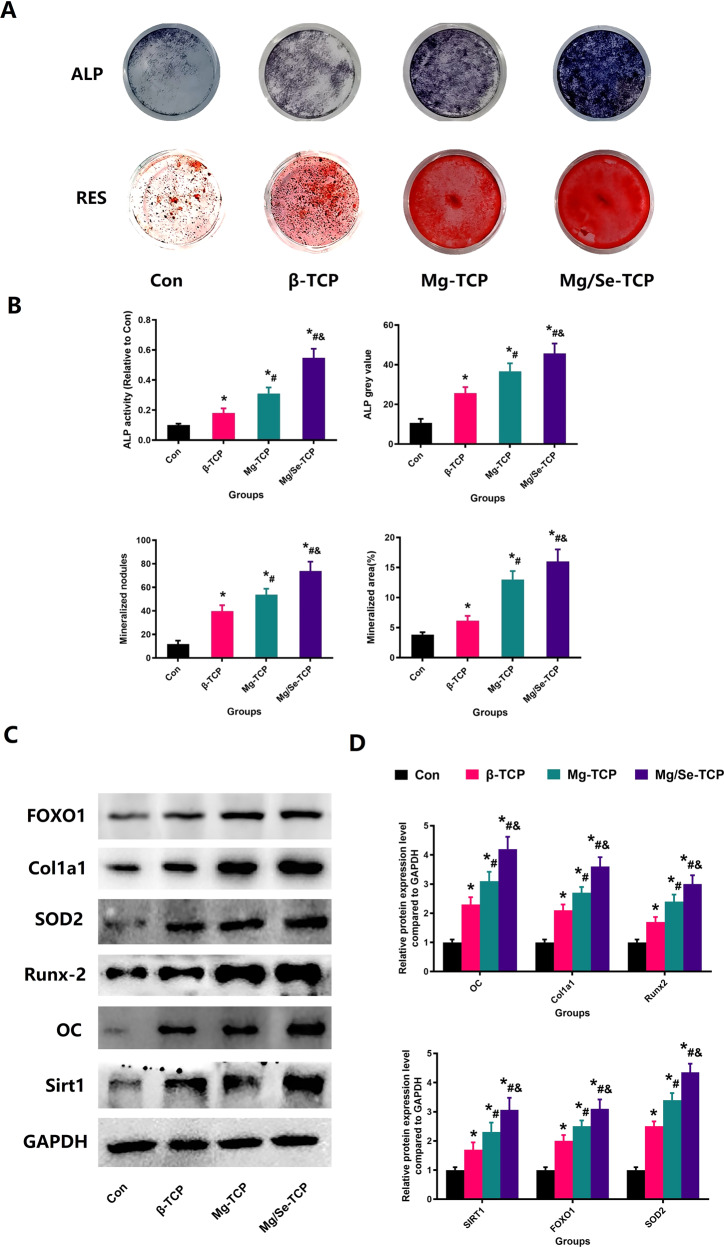
Mg/Se-TCP treatment could increase the osteogenic potential of MC3TE-E1. Representative pictures of ALP staining and RES staining in the general view (**A**), Quantitative results of ALP Staining and Alizarin Red Staining including nodules, mineralized area, ALP activity, and ALP gray value (**B**), Representative pictures of western blot results (**C**), and target protein expression (**D**) after treatment with different intervention options. *N* = 5 specimens/group. *Vs. Con group, *P* < 0.05, ^#^Vs. β-TCP group, *P* < 0.05, &Vs. Mg-TCP group, *P* < 0.05

In the vitro study, the effect of β-TCP, Mg-TCP, and Mg/Se-TCP on osteogenesis of MC3T3-E1 were evaluated by ALP staining and RES staining (Fig. 8). After culturing for 14 and 21 days, the ALP staining area and formation of red calcium nodules inβ-TCP, Mg-TCP, and Mg/Se-TCP groups were observed to increase, compared with the Con group. Moreover, the ALP staining area and formation of red calcium nodules were improved under Mg/Se-TCP treatment, compared with the β-TCP and Mg-TCP groups. Once again, the quantitative results of RES staining and ALP staining presented as ALP gray value, ALP activity, mineralized area and mineralized nodules demonstrated that the osteogenesis of MC3T3-E1 was partially restored under Mg/Se-TCP treatment.

The WB results of target protein expressions, including FOxO1, SIRT1, SOD2, Runx-2, OC, and Cola1a of the β-TCP, Mg-TCP, and the Mg/Se-TCP groups were significantly lower than that of the Con group (*P* < 0.05). In addition, treatment with Mg/Se-TCP could enhance the levels of FOxO1, SIRT1, SOD2, Runx-2, OC, and Cola1a (*P* < 0.05), compared with the β-TCP and Mg-TCP group.

### The changes in cell viability, ROS and SIRT1, and SOD2 expression

**Fig. 9:**
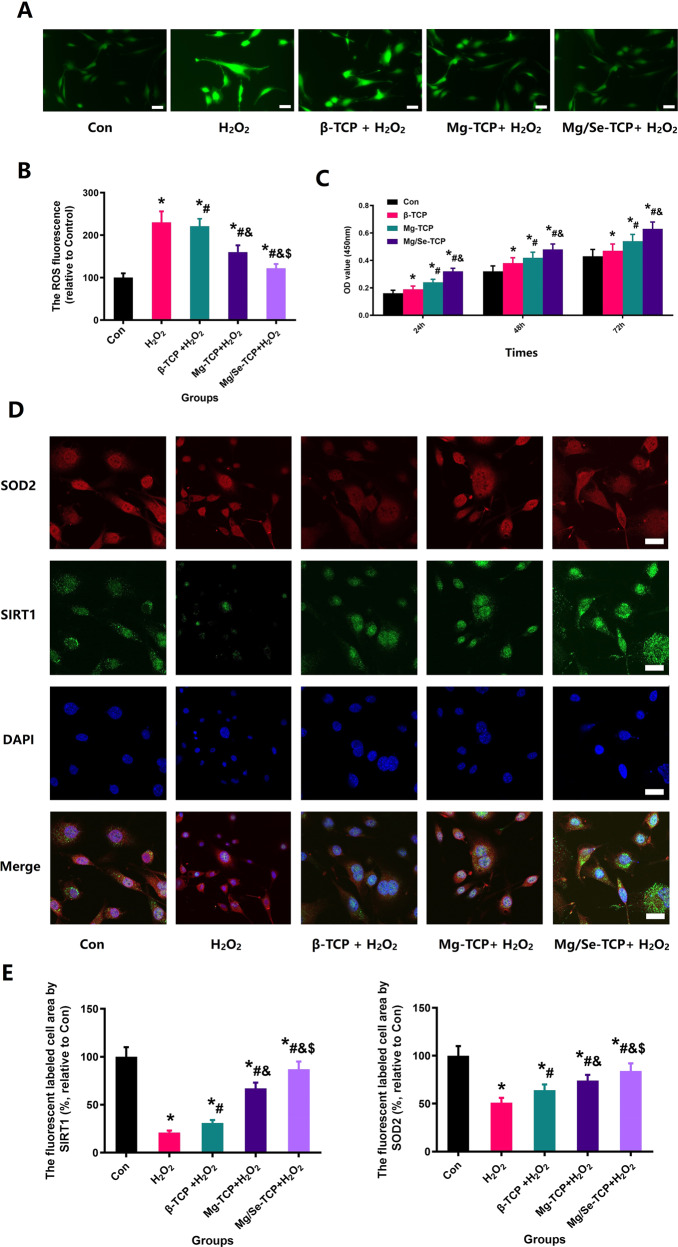
Mg/Se-TCP can significantly decrease MC3T3-E1 cellular ROS levels and improve the expression of SIRT1 and SOD2. **A** Representative pictures for intracellular ROS level of MC3TE-E1 under different treatments; Scale bar: 25 µm; **B** The quantitative results of ROS level; **C** Results of the CCK-8 test for the viability of MC3TE-E1 with different treatments. **D** Representative pictures for intracellular SIRT1 and SOD2 expression of MC3TE-E1 Cells; Scale bar = 25 µm; **E** Quantitative results of intracellular SIRT1 and SOD2 expression. *Vs. Con group, *P* < 0.05, ^#^Vs. H_2_O_2_, *P* < 0.05, ^#^Vs. H_2_O_2_ + β-TCP group, *P* < 0.05, &Vs. H_2_O_2_ + Mg-TCP group, *P* < 0.05

The results of cellular ROS levels are shown in Fig. 9A. ROS levels increased significantly after H_2_O_2_ intervention, while cellular ROS levels significantly decreased after H_2_O_2_ + Mg/Se-TCP intervention, compared with H_2_O_2_ + β-TCP, H_2_O_2_ + Mg-TCP, and H_2_O_2_ groups. Moreover, treatment with β-TCP, Mg-TCP, and Mg/Se-TCP could enhance the cell viability at 24, 48, and 72 h after H_2_O_2_ intervention (*P* < 0.05). The quantitative results are shown in Fig. 9B. ROS was significantly reduced by the intervention of β-TCP, Mg-TCP, and Mg/Se-TCP (*P* < 0.05). To more intuitively observe the expression of SIRT1 and SOD2, immunofluorescence was performed on the MC3TE-E1 under different interventions. The representative immunofluorescence images (Fig. 9D) and quantitative results of SIRT1 and SOD2 expression are shown in Fig. 9E. The expression of SIRT1 and SOD2 decreased significantly after H_2_O_2_ intervention, while the SIRT1 and SOD2 levels significantly increased after H_2_O_2_ + Mg/Se-TCP intervention, compared with H_2_O_2_ + β-TCP, H_2_O_2_ + Mg-TCP, and H_2_O_2_ groups (*P* < 0.05).

## Discussion

Owing to the impaired bone formation caused by estrogen deficiency, bone healing is obviously delayed or impaired in postmenopausal women with osteoporosis and the experimental OVX model [35, 36]. Despite a large number of different tissue engineering materials for bone repair developed rapidly, therapy for bone defects remains a major challenge in the field of bone implantology for clinical applications. Based on superior osteoinduction and osteogenesis, we hope to improve bone regeneration through the incorporation of Mg and Se by stimulating the repair ability of β-TCP to meet expectations. Our research observed and confirmed that the application of Mg/Se-TCP could acquire satisfied regeneration of a critical size defect in the femoral metaphysis of OVX rats.

In the present study, we report that promise of using 3D-printed β-TCP could promote bone regeneration in OVX rats. Moreover, we used computer-assisted 3D-printing to fabricate β-tricalcium phosphate scaffolds, because of the biomaterials with controllable pore structure and improved mechanical strength [12–14]. Although the 3D-printed β-tricalcium phosphate scaffolds used in this study can promote the healing of bone defects, a large area of the defect is still visible for 12 weeks. Therefore, this healing approach could not necessarily meet the expectations of physicians and patients due to large unrepaired areas still existing through imaging and histology evaluation, as shown in previous studies [11, 37], which presents us with new demands and challenges. In this study, a large amount of bone tissue formed in the defected areas was observed in the Mg-TCP group. Although the addition of Mg can be observed to enhance the bone regeneration ability of β-TCP, we still found that 3% Mg-modified β-TCP could not complete bone defect repair at 12 weeks. Excitingly, we observed almost completely repaired bone defects in the Mg/Se-TCP group, indicating that the incorporation of Mg and Se significantly enhanced the local osteogenic environment and finally achieved satisfactory results.

Owing to the function and activity of osteoblasts playing a pivotal role in bone regeneration, MC3T3-E1 research was carried out further. As we hypothesized, the activity, ALP expression and mineralization of MC3T3-E1 in the Mg/Se-TCP groups were discovered at higher levels, when compared with the Con group and the β-TCP group and Mg-TCP group, which was verified by ALP staining and RES staining and CCK-8 detection. Due to the increased ROS production in the development and progression of osteoporosis, studies have indicated that the increased levels of oxidative stress induced by estrogen deficiency is a major pathogenic factor of restricted bone formation and related cell injury [38, 39]. As remarkable indexes, the levels of SOD2 can sensitively reflect the condition of oxidative stress [40, 41]. Sirtuin1 (SIRT1), a nicotinamide adenine dinucleotide-dependent deacetylases, could regulate the activity of downstream target genes and prevent bone loss in an animal model [42, 43]. FoxO1, as a downstream gene of SIRT1, can be activated by oxidative stress to promote the expression of downstream antioxidant genes to reduce oxidative stress to protect cell normal metabolism [44, 45]. The previous study has shown that SIRT1 could directly deacetylation FOXO3A and induces antioxidant enzyme expression, including SOD2 [46]. In addition, the intracellular ROS level of MC3TE-E1 Cells more intuitively shows the changes of ROS in different groups. Moreover, WB observed that the expression of SIRT1, FoxO1 and SOD2 increased significantly were observed in the Mg/Se-TCP group. In the study, we have also observed that the levels of SOD2 and SIRT1 detected by immunofluorescence from MC3T3-E1 were significantly reduced after H_2_O_2_ intervention, and the expression of SOD2 and SIRT1 of MC3T3-E1 were reversed after Mg/Se-TCP treatment.

Based on the above results, we deduced that estrogen deficiency can inhibit the activity and function of osteoblasts by disrupting the balance of oxidative stress in osteoblasts, causing limited bone formation and poor regeneration. In view of the above results, we could conclude that estrogen deficiency can upregulate the level of oxidative stress in osteoblasts, resulting in decreased osteogenic capacity, which is manifested as decreased ALP expression and reduced mineralization function and impaired bone formation capacity, which eventually led to unsatisfied results of bone regeneration in OVX rats. Moreover, the incorporation of Mg and Se can restore the oxidative stress balance of osteoblasts and protect the activity and function of osteoblasts, thereby improving osteogenic ability. In addition, due to the enhancement of bone formation ability and the increase of material degradation ability, it leads to local calcium and phosphorus enrichment, creating a better environment for bone formation. Eventually, the phenomenon appeared and observed in this study that the defect in the Mg/Se-TCP group was repaired quickly and with high quality.

## Conclusion

In summary, our research results confirm that osteoporosis impaired the activity and function of MC3T3-E1 and inhibits bone regeneration by up-regulating intracellular ROS levels. Mg/Se-TCP can improve the activity and function of osteoblasts and enhance bone regeneration mediated by reducing intracellular ROS in OVX rat models. However, the absence of a normal control group and Se-TCP group, only the timing point of 12 weeks and the use of different Mg and Se contents to observe the treatment effect brought shortcomings to the study, which will be further explored at a later stage.
